# Addressing Policy Coherence Between Health in All Policies Approach and the Sustainable Development Goals Implementation: Insights From Kenya

**DOI:** 10.34172/ijhpm.2020.212

**Published:** 2020-11-16

**Authors:** Joy Mauti, Lara Gautier, Faith Agbozo, Veronica Shiroya, Nasreen S. Jessani, Jale Tosun, Albrecht Jahn

**Affiliations:** ^1^Heidelberg Institute of Global Health, Heidelberg University, Heidelberg, Germany.; ^2^Département de Gestion, Évaluation et Politique de Santé, École de Santé Publique, Université de Montréal, Montreal, Canada.; ^3^Department of Sociology, McGill University, Montreal, Canada.; ^4^Department of Family and Community Health, School of Public Health, University of Health and Allied Health Sciences, Ho, Ghana.; ^5^Department of International Health, Johns Hopkins Bloomberg School of Public Health, Baltimore, MD, USA.; ^6^Center for Evidence Based Health Care, Department of Global Health, Stellenbosch University, Stellenbosch, South Africa.; ^7^Africa Centre for Evidence, University of Johannesburg, Johannesburg, South Africa.; ^8^Institute of Political Science, Heidelberg University, Heidelberg, Germany

**Keywords:** Health Policy, Intersectoral Collaboration, Sustainable Development Goals, LMICs, Sub-Saharan Africa, Kenya

## Abstract

**Background:** Addressing health in the Sustainable Development Goals (SDGs) calls for intersectoral strategies that mutually enhance both health promotion and sustainable development. Health in All Policies (HiAP) approach aims to address this as well as promote ownership among key stakeholders. Kenya was at the forefront of adopting the SDGs and has committed to the HiAP approach in its Health Policy document for the period 2014-2030. This study aims to assess how the adoption of the HiAP approach can leverage on SDGs implementation in Kenya.

**Methods:** This is an exploratory case study using qualitative data and some descriptive quantitative data. The Organisation for Economic Co-operation and Development’s (OECD’s) eight building blocks for policy coherence on sustainable development was our guiding framework. Qualitative data was derived from a review of relevant peer-reviewed and grey literature, as well as 40 key informant interviews and analyzed in NVIVO. Quantitative data was accessed from the United Nations SDG indicator database and exported to Excel.

**Results:** Kenya has expressed a strong political commitment to achieving the SDGs and has now adopted HiAP. The study showed that Kenya can leverage on local level implementation and long-term planning horizons that it currently has in place to address the SDGs as it rolls out the HiAP approach. The SDGs could be mapped out against the sectors outlined in the Adelaide statement on HiAP. It is also possible to map out how various ministries could coordinate to effectively address HiAP and SDGs concurrently. Funding for HiAP was not addressed in the OECD framework.

**Conclusion:** Kenya can advance a HiAP approach by leveraging the ongoing SDGs implementation. This will be made possible by facilitating coordinated intersectoral action both at national and local level. Funding for HiAP is crucial for its propagation, especially in low- and middle-income countries (LMICs) and can be considered in the budgetary allocations for SDGs.

## Background

**Key Messages**

**Implications for policy makers**

- Policy-makers can utilize the proposed mapping of Sustainable Development Goals (SDGs) against the sectors outlined on the Adelaide statement on Health in All Policies (HiAP) to facilitate the HiAP approach adoption through SDG implementation.
- Policy-makers can promote creation or utilization of existing intersectoral groups and governance structures at local level for both HiAP and SDGs.
- Policy-makers, especially from low- and middle-income countries (LMICs), can use the Organisation for Economic Co-operation and Development’s (OECD’s) framework to assess policy coherence between health promotion strategies and sustainable development.

**Implications for the public**

This research shows how we can harmonize global policies with local realities -especially in a resource-limited setting. It shows the vital role local level structures and advocacy to promote policy adoption and implementation. This is particularly important in the case of Health in All Policies (HiAP) and Sustainable Development Goals (SDGs) implementation, as elaborated in this study.

###  The Linkage Between the Sustainable Development Goals and the Health in All Policies Approach

 In September 2015, the United Nations member states adopted Agenda 2030 within which the 17 Sustainable Development Goals (SDGs) are supposed to be “*integrated and indivisible*,” therefore complementary in nature.^1^ This calls for a systematic approach when evaluating the SDGs’ synergies and trade-offs.^2^ While SDG 3 aims to “*ensure healthy lives and promote wellbeing for all at all ages*,” core health targets are either embedded in other goals or influenced by them.^2-5^ Addressing these goals in order to promote health will require new ways of working, and ensuring stakeholder engagement.^4-6^

 There are several encouraging strategies that have been considered in order to seek to advance health from an intersectoral perspective. For instance, the 2016 Shanghai declaration on health promotion intends to foster the interconnectedness of health and all SDGs.^7^ It also calls for political will to strengthen policy coherence for improved health equity and economic development.^4^ Health in All Policies (HiAP) is one such approach.

 HiAP is defined as, “*an approach to public policies across sectors that systematically takes into account the health implications of decisions, seeks synergies, and avoids harmful health impacts in order to improve population health and health equity.*”^8^ As such, HiAP is a whole-of-government approach promoting all sectors to have a consideration for health.^8^ The HiAP approach also emphasizes the need for collaborative leadership within and between governments.^8,9^

 In 2017, 150 HiAP experts and practitioners representing 21 countries congregated in Adelaide, Australia and committed to fulfil the Shanghai Declaration^10^. In addition, in its ^13th^ General Programme of Work, 2019–2023, the World Health Organization (WHO) stated that, “*Multisectoral action becomes possible when health actors are empowered to effectively engage in and support policy processes in other sectors. WHO will promote ‘Health in All Policies’ and governmental cabinet approaches to cross-sectoral action and policy coherence.*”^11^ The 2019 World Health Assembly resolution A72/11 also reiterates the importance of a coordinated intersectoral action and especially HiAP in implementing the SDGs.^6^

 Given clear resonance between the SDG intentions and the HiAP approach, the SDGs provide a unique opportunity to advance HiAP.^12,13^ Ramirez-rubio et al in 2018 outlined some of the opportunities SDGs present in addressing some challenges that affect HiAP as shown in Table 1.

**Table 1:** Examples of HiAP Challenges Addressed Within the SDGs

**HiAP Challenges**	**Opportunities Through the Implementation of SDGs**
Disparities between political and technical health aspects.^14-16^	The SDGs encourages holistic thinking in governance to address both technical and political processes.^17^
Lack of HiAP awareness outside the health sector.^18-20^	The SDGs are universally accepted; therefore, can raise awareness for HiAP.^19^
HiAP falling off the political agenda over time.^17^	
Limited research on funding and governance mechanisms for HiAP.^17,21^	“Avalanche” of research addressing funding and governance mechanisms for SDGs.^22^
Difficulty in sustaining partnerships due to lack of evidence on association between different sectors.^15,17-19,23,24^	The SDGs can inform development of a HiAP framework as they address all sectors that influence health.

Abbreviations: SDGs, Sustainable Development Goals; HiAP, Health in All Policies. Source: Ramirez-Rubio et al.^25^

###  Health in All Policies and Sustainable Development Goals in Kenya

 Literature on health-related intersectoral action and SDGs is sparse in low- and middle-income Countries (LMICs).^26-28^ However, several studies do cover African policy-makers’ views on how to achieve the SDGs.^29-31^ Kickbusch et al in 2017 highlighted South Sudan, Namibia and Zambia as examples of African countries that have adopted a HiAP approach – although still in early stages – to advance the SDGs.^4^

 Kenya is committed to the HiAP approach as per its sixth policy objective in the Kenya health policy document for 2014-2030 which aims to “*strengthen collaboration with private and other sectors that have an impact on health*” and explicitly addresses various social determinants of health through HiAP.^32^

*“The policy will also seek to influence the following social determinants of health: women’s literacy, access to safe water and adequate sanitation, nutrition, safe housing, occupational hazards, road safety, security, income, and community participation, among others.*”^32^

 Scholars note that the adoption of the HiAP approach in Kenya can be an important “win-win” approach for Kenya by maximizing on its policy coherence with the SDGs.^33,34^ This empirical study thus contributes to the literature by specifically addressing how HiAP adoption can leverage implementation of the SDGs in Kenya.

###  Study Aims

 The study had several aims:

1. Understand how a HiAP approach can leverage development agendas promoting the SDGs.
2. Assess how both the HiAP approach and the SDGs’ implementation were articulated in Kenyan policy documents, and how they are perceived by the various stakeholders.
3. Offer insights into the opportunity of developing a monitoring and reporting framework for Kenya’s HiAP approach using SDG indicators.

###  Conceptual Framework: OECD’s Policy Coherence for Sustainable Development 

 To assess the congruence between HiAP and SDGs in Kenya, we perused the Organisation for Economic Co-operation and Development’s (OECD’s) Policy Coherence for Sustainable Development (PCSD) framework, which was developed in 2016.^35^ According to the PCSD Framework, policy coherence is defined as, “*the process of fostering synergies across economic, social and environmental policy areas; identifying trade-offs and reconcile domestic and international objectives; and addressing the spill-overs of domestic policies on other countries and on future generations.*”^35^ Figure shows the PCSD framework 8 building blocks which are in line with the SDG Target 17.14 that addresses PCSD.^35^ This framework helps us to explore the economic, political and social areas that are important for both HiAP and SDGs. The PCSD building blocks also address very similar thematic areas that Ramirez-Rubio et al proposed as areas of linkages between HiAP and SDGs.

**Figure:**
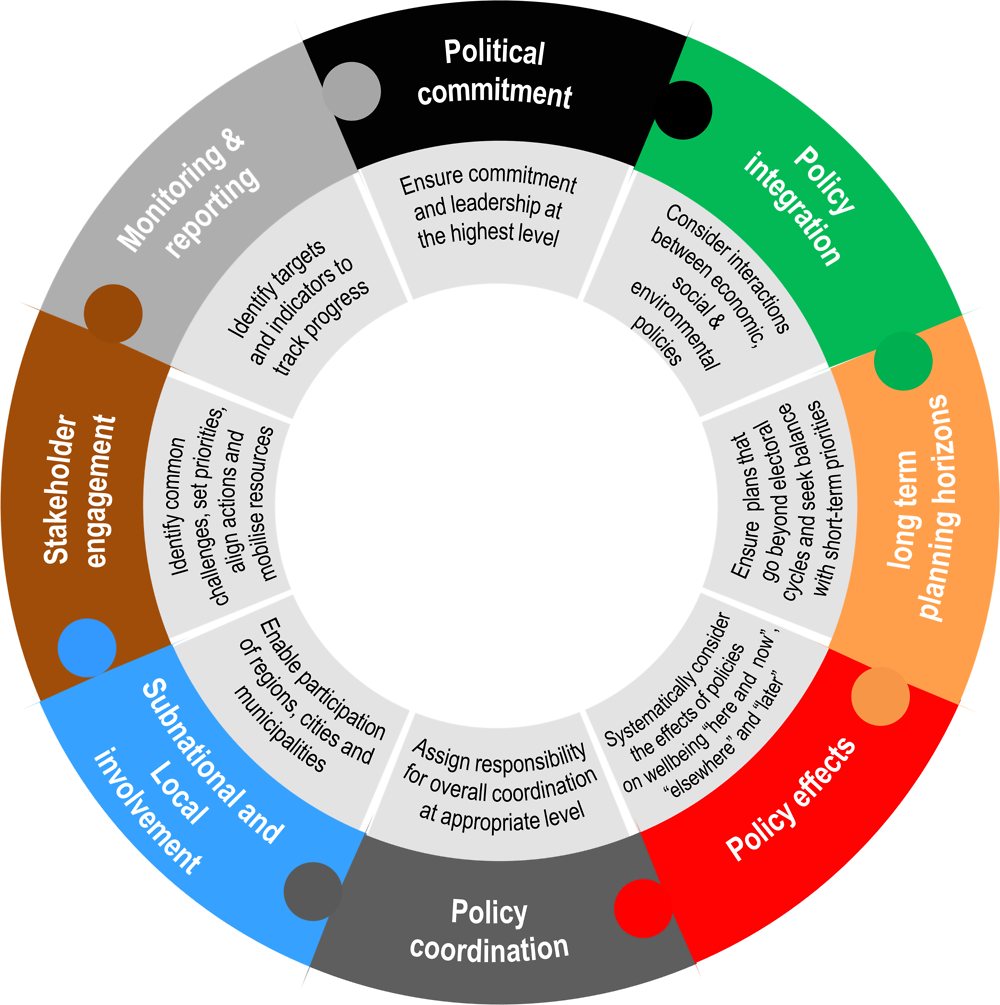
PCSD Building Blocks. Abbreviation: PCSD, policy coherence for sustainable development. Source: OECD.^35^

 The framework above highlights critical aspects with respect to (*a*) political commitment, (*b*) the importance of inclusion (stakeholder engagement, subnational and local involvement), (*c*) Policy coherence (coordination, integration and effects), (*d*) long term planning, and (*e*) Monitoring and reporting. Throughout these building blocks there is an emphasis on intersectoral collaboration (ISC), multi-stakeholder engagement, and accountability which provides a formidable foundation for assessing the congruence of HiAP approach with the SDGs as seen in Table 2.

**Table 2:** Operationalization of the OECD Building Blocks for Policy Coherence Between HiAP and SDGs

**Aim **	**Building Blocks **
How HiAP can leverage on the development agendas that address SDGs	Political commitment Long-term planning horizons Subnational and local involvement
How HiAP adoption and implementation can progress alongside SDGs implementation	Policy Integration Policy coordination Institutional coordination Stakeholder engagement
Assess whether or not a monitoring and reporting Framework for HiAP can be developed using SDG indicators	Monitoring and reporting Policy effects

Abbreviations: SDGs, Sustainable Development Goals; HiAP, Health in All Policies; OECD, Organisation for Economic Co-operation and Development.

## Methods

###  Study Design

 This is an exploratory case study using qualitative data and some descriptive quantitative data.^36,37^ The case being studied is the HiAP approach adoption in Kenya, a LMIC, in the context of SDGs implementation. According to Stake’s typology, a case study is important because it enables us to delve into the “*particularity and complexity of a single case*,” thus enabling us to better consider the peculiarities of the case.^36,37^ Data sources for qualitative analysis included peer-reviewed and grey literature as well as key informant interviews. The United Nations SDG indicator database for Kenya was used to access the quantitative data reported in this study.

###  Study Setting

 Kenya is categorized as a lower middle-income economy with 36.1% of the population living in extreme poverty (surviving on less than $1.90 a day) in 2015/2016.^38^ The Kenyan population also suffers an uneven access to healthcare.^39^ In an attempt to address a highly centralised and hierarchical government which was affected by corruption, the government effectively engaged in a process of decentralization following the promulgation of the 2010 Constitution of Kenya.^40,41^ Devolution – one form of decentralization – resulted in one National Government and 47 decentralized governments.^42,43^

 According to the constitution, the two levels of governments are interdependent and undertake their relations through consultation and cooperation.^44^ This cooperation is supposed to promote formulation and implementation of socio-economic policies.^40,45^ The central government retains policy development and management of overall national and international affairs.^45,46^ The county governments oversee policy implementation within their designated geographical location.^47^ For SDG implementation, counties develop their own county integrated development plans (CIDPs) which must align with Vision 2030 Medium-Term Plans (MTPs) in that given period.^41^Table 3 summarizes Kenya’s lead institutions as well as key policy documents for SDGs.

**Table 3:** Kenya’s Key SDG Institutions, Actors and Policy Documents

**Administrative Level**	**Lead Institutions**	**Policy Documents**	**Legality**
National	Ministry of Devolution and Planning - SDGs coordinating department	Vision 2030 (2007) MTP 3 2018-2022 (2018)	Binding Binding
County	Office of Director of planning and economic affairs Council of Governors	CIDPs* (various dates)* County of governors committee work plans *(various dates)*	Binding Binding

Abbreviations: SDGs, Sustainable Development Goals; CIDPs, county integrated development plans; MTPs, medium-term plans.

###  Data Collection 

####  Literature Review

 Peer-reviewed literature was retrieved from PubMed database using a combination of keywords related to HiAP and SDGs. Google Scholar and Google were perused for both peer-reviewed as well as grey literature searches as seen in Table 4.

**Table 4:** Overview of the Literature Review and Selection Process

**Selection Process**	**Peer-Reviewed Literature**	**Grey Literature**
Language	English	English
Search Terms	PubMed: “Health in All Policies,” “Health in All Policies” AND Development, “Health in All Policies” AND SDGs, “Health in All Policies” AND policy coherence, SDGs AND policy coherence.	Google Scholar: HiAP and SDGs, Policy coherence and SDGs, Trade-offs between SDGs, Synergies between SDGs, HiAP and policy coherence. Google: Kenya and SDGs, Kenya and HiAP, Kenya Vision 2030, Kenya CIDPs, Kenya and Intersectoral governance.
Publication type	Original research, all kinds of reviews and commentaries.	Websites, reports, and any content related to HiAP and SDGs in Kenya.
Documents selected	Out of the total 400 results 50 were selected. 54 other relevant papers were identified (N = 104).	National policy documents = 57. National documents obtained from interviewees = 3. International documents = 31 (N = 91).
Grand total	195 documents	

Abbreviations: SDGs, Sustainable Development Goals; HiAP, Health in All Policies; CIDPs, county integrated development plans.

####  Interviews

*Interviewees selection*: Purposeful and snowball sampling approaches were used to select key informants. The first author, who is Kenyan, approached all Kenya’s government ministries – either by email, telephone, or physically at ministry offices – from August 2016 to March 2017 to recruit interviewees. Formal requests were addressed to the office of the permanent secretary in every ministry. From there, the interviewer was directed to the designated interviewee. The interviewees were either working at the highest level of policy-making or working at technical level in the ministry.

*Participant overview: *16 out of the total 20 ministries in Kenya in 2016/2017 were contacted and agreed to have representatives interviewed. Representatives from the government were all from the national level and ranged in positions from Under-Secretaries to Economists, Policy Directors, Head of Departments and Department members. In addition, a list of potential key informants from outside the ministries was generated and snowball sampling was used to contact representatives from development partners, civil society, academia and policy institutes. In total 24 government officials, 6 development partners, 2 Heads of NGO Consortiums, 1 NGO, 3 academic professors, 2 policy analysts and 2 independent consultants were interviewed. Most of the interviewees outside the health sector were not aware of HiAP approach but were very familiar with the concept of ISC and that is what was leveraged on.

*Instrument development and use*: Interview guides used to obtain data from key informants in government and non-government sectors were developed and tested. The questions explored interviewees’ knowledge of HiAP or ISC; their knowledge and perception of Kenya’s involvement in MDGs and SDGs, thoughts on the role of HiAP/ISC in relation to the SDGs for HiAP, and economic consideration for HiAP/ISC – whether it was profitable or not to adopt this approach and Vision 2030. Although the OECD analytical framework was adopted after the interviews had been done, there were some similarities between the questions asked and the OECD building blocks, especially political commitment, policy integration, policy and institutional coordination and stakeholder engagement. Interviews were conducted in English with extensive notes taken. All but 4 provided consent to be audio recorded Extensive notes were taken in all interviews.

####  United Nation Sustainable Development Goal Indicators Database

 To assess whether a monitoring and reporting framework could be developed, all the relevant SDGs were mapped out against the social determinants Kenya highlighted under the HiAP policy objective. We then assessed the United Nations global SDG indicator database to retrieve data for Kenya by first selecting the indicator under the given target and goal, then the geographical location, the years from 2015 onwards and finally by downloading the data as an excel sheet.^48^

###  Data Analysis

 All the documents retrieved were uploaded in an Endnote file. They were then screened for content specifically related to our thematic area, and extracted relevant data onto Word documents which were uploaded to NVIVO 12 for further analysis. We then discussed the results obtained for Kenya with regards to the peer-reviewed literature. Budget analysis was done by reviewing all the 47 CIDPs budgets in detail to see how the counties allocated funds for their stated projects, and whether or not the allocation was per the SDGs. The main policy documents pertaining to health and SDGs reviewed in Kenya are listed in Table 5.

**Table 5:** Reviewed National Policy Documents and Reports (Grey Literature)

**Title**	**Addressing SDGs or HiAP?**	**Publication Year**	**Reference**
Implementation of the Agenda 2030 for Sustainable Development in Kenya	SDGs	2017	^49^
Guidelines for preparation of county Integrated development plans (revised)	SDGs	2017	^50^
Vision 2030’s Medium Term Plan as a Framework for Implementation of the Sustainable Development Goals	SDGs	2016	^51^
Voluntary national review of progress on SDGs in Kenya	SDGs	2017	^52^
Sustainable Development in Kenya: Stocktaking in the run up to Rio+20	SDGs/HiAP	2012	^53^
Kenya Vision 2030: The Popular Version (2007)	SDGs/HiAP	2007	^54^
Vision 2030 Third Medium Term Plan 2018-2022	SDGs/HiAP	2018	^55^
Kenya Health Policy 2014-2030	HiAP	2014	^32^
Addressing the Social Determinants of Health in Kenya: Framework for Health in All Policies and Inter-sectoral Action	HiAP	2013	^56^
Reforming health care in Kenya: prospects for health-in-all policies approach	HiAP	2011	^57^
Review of social determinants of health and health indicators in Kenya	HiAP	2013	^58^
County Integrated Development Plans	SDGs/HiAP	2020	^59^

Abbreviations: SDGs, Sustainable Development Goals; HiAP, Health in All Policies.

 All interviews were transcribed and uploaded into NVIVO 12, and then analyzed using a qualitative framework analysis which is a procedure to assess qualitative data by sorting and charting it in accordance with key issues and themes in the given research study.^60,61^ A deductive approach was used, whereby the OECD framework on policy coherence’s 8 building blocks guided the coding and analysis. The blocks served as the major themes of the codebook. Starting from these themes, we coded the material ie, interview transcripts, literature in NVIVO using an iterative approach whereby subthemes and codes were identified and refined as we progressed through the analysis.

## Results

 The results report interviewees’ perspectives on HiAP and SDGs implementation as well as data from literature and the SDGs database. The results have been presented in accordance to the 8 PCSD building blocks under the 3 objectives of the study.

###  Understand How a HiAP Approach Can Leverage Development Agendas

####  OECD Building Block: Political Commitment

 Political commitment and leadership at the highest level of government is essential for both HiAP and SDGs. Kenya’s strong commitment to both the SDGs and Africa Union’s Agenda 2063 has been facilitated by political leadership and an Inter-Agency Technical Working Group which was established in early 2015.^52^ This Working Group comprises representatives from all the ministries, Kenya’s National Bureau of Statistics, National Council for Population and Development, civil society, and the private sector.^49^

 Political commitment for HiAP in Kenya is evidenced by it being outlined as a policy objective in the Kenya Health Policy 2014-2030. As confirmed by one interviewee, the HiAP approach was proposed by a former Minister of Health during her tenure as one of the commissioners of the global commission for social determinants of health.

 As the Ministers of Health and other health professionals are part of the Inter-Agency technical working group, they have an opportunity to propagate for HiAP in these meetings as an approach that encourages holistic thinking in governance.

####  OECD Building Block: Long-term Planning Horizons

 Vision 2030 has been divided into multiple 5-year MTPs. MPT3 (2018-2022) and MTP4 (2023-2028) are expected to mainstream the SDGs.^49,52,62^ In addition, Kenya has to honor regional commitments, in particular the African Union’s Agenda 2063 which is this continent’s blueprint for development for the period 2013 to 2063.^51,54,62^ Through its long-term outlook, 17 interviewees indicated that Vision 2030 supersedes the political challenges of electoral cycles and personal whims of politicians and ensures that every new party maintains their obligation to it.

*“What I would say is that Vision 2030 and SDGs are very good because they enable us implement what every government is planning and being a long-term kind of planning blueprint, it is able to avoid politics. So, any political party that will come to run the country finds Vision 2030 is there being implemented, finds the SDG targets are there – they are set. What you add into it, they are just like flavour you come with in your manifesto” *(KI39 – Government official).

 Ten interviewees indicated that Vision 2030 tries to bring all development aspects that are linked to health.

*“[Vision 2030] is the only thing that tries to bring together all the ingredients of health for all, but it is difficult to define how this happens in the Kenyan scene. You can only point to Vision 2030 and say – looking at the social pillar, the economic pillar, socio-economic pillars – That is where you can see some kind of concerted effort to look at the health in all policies” *(KI13 – Independent Consultant).

 The current Health Policy document has the same timeline as the Vision 2030 which provides an opportunity for HiAP to be mainstreamed simultaneously with the SDGs.

####  OECD Building Block: Subnational and Local Involvement

 An SDGs Liaison office within the secretariat of the Council of Governors for the counties has been created in order to facilitate coordination between the national and the county government. Specifically, for ISC, 14 interviewees indicated that the devolved system of government posed an opportunity for its implementation at the county level.

*“I would say this: at the county level, these divisions we have at national level begin to fade out, because we sort [of] have like these divisions and division heads who it is really easy for them to sit [together]. The guy in the ministry of water or the ministry of education for them to sit on the same table with someone from the ministry of health is really easy because it a smaller area of operation” *(KI40 – NGO representative).

 Three interviewees talked about multi stakeholder health promotion structures called the Health Promotion Advisory Committee (HPAC). They also stated that it was important for the establishment of HPACs both at national and county level.

*“There were a number of recommendations and one was to have a HPAC at the national level and at the county level because after devolution, we realize that there are some policies which would be made at the county level. So, it would not be enough to have a committee at the national level, we still need some platforms at the county level so that whatever policies or whatever communication are developed at national level they can have a platform at county level to be discussed before it goes to the community” *(KI18 – International NGO Representative).

 HPACs consist of members from major economic sectors, religious leaders, and “village champions” who are tasked with advising the county on health issues. The HPACs are led by the County Health Promotion officer have several responsibilities: advocacy on resources and policy; lobbying for funds; and disseminating policy communications from the national level to the county level. HPAC members translate these policy communications, if possible, into the national language (Kiswahili) or into local languages. At the time of this study, there were 33 out of 48 possible HPACs (47 at the county level and one at the national level) in place.

 HPACs offer an opportunity for the HiAP approach to be established at local level. The HPACs could collaborate with the SDGs Liaison office at the as they already have a diverse set of representatives who can leverage on health promotion to be a central focus in sustainable development.

###  HiAP Adoption and SDGs Implementation in Kenya

####  OECD Building Block: Policy Integration

 Policy integration ensures that there is a balance in addressing the economic, social and political dimensions of development.^35^ While Vision 2030 addresses all 3 dimensions, 4 interviewees indicated that some sectors received more attention than others.

*“One of its weaknesses is that it [Vision 2030] is very ambitious. It requires a lot of revenue generation from the government and that didn’t happen and so that means of course some sectors in my view are starved of the resources” *(KI7 – Government Official).

 Policy integration also aims to ensure that the global agenda is addressed at regional, national and local levels within a nation.^35^ The current MTP3 for the period 2018-2022 which informs the second generation of CIDPs explicitly shows how the SDGs will be implemented but only mentions that it is aligned with the 7 aspirations of the Agenda 2063.^55^ A study conducted by the SDGs Kenya forum found out that it was a challenge for some goals and targets to fit in properly within Vision 2030’s MTPs which inform the CIDPs.^51^ These were the goals addressing multilateral partnerships, development assistance, immigration and regional integration, which will not be easy to address, especially at the county level.^51^

 All 47 CIDPs were reviewed with 44 county CIDPs mentioning links to Agenda 2030 and 30 county CIDPs mentioning links to Agenda 2063. Only 3 CIDPs directly addressed each SDG. Given that budgets are considered an essential tool for policy integration, all 47 CIDP budgets were reviewed.^35^ Only one county in one draft of their CIDP showed how they allocate funds per SDGs.^63^

 Therefore, the sectors under HiAP could be integrated in the Vision 2030 as they both address the economic, political and economic aspects of development. This can also assist in equitable focus on the sectors especially in the budgetary allocations. Additionally, by collaborating with the SDG liaison office, the HPACs have better chances to lobby for budgetary allocations for health as opposed to competing for the funds.

####  OECD Building Blocks: Policy and Institutional Coordination and Stakeholder Engagement

 In Kenya, the Ministry of Devolution and Planning coordinates SDG implementation across the ministries and at the county level. There is a specific SDGs office within the ministry that facilitates both government and non-government stakeholders’ engagement. This SDGs office also works with the Inter-Agency Technical Working group. Outside the government, there is the SDG Kenya forum, which was initiated by the civil society in Kenya. The forum not only coordinates civil society engagement in the SDGs but acts as the liaison between the civil society and the government. All interviewees agreed that the implementation of the SDGs is a great opportunity to improve ISC at all government levels.

*“The intersectoral collaboration is very key in terms of helping Kenya [to] achieve the SDGs. That synergy is very key because it is the coordination framework that will help to midwife [facilitate] those results and I think there is commitment from stakeholders especially in government to ensure that that collaboration continues and is strengthened” *(KI32 – Development Partner).

 Ministry of Health could leverage these established structures and collaborations at all government levels and beyond to not only create awareness but also establish HiAP. Table 6 illustrates an example of how ministries could collaborate or take up leadership roles in facilitating ISC to address both HiAP and SDGs in Kenya.

**Table 6:** Suggested Linkages Between Social Determinants of Health in the Current Kenya Health Policy 2014-2030 and the Relevant SDGs

**Sectors According to Adelaide Statement**	**Kenya’s Health Policy Social Determinants of Health focus **	**SDGs**	**Example of Collaborating/Leading Ministries**	**Examples of Cross Cutting Ministries**
Education and early life	Women’s literacy	SDG 4: Ensure inclusive and equitable quality education and promote lifelong learning opportunities for all	Ministry of Education Ministry of Public Service, Youth & Gender Affairs Ministry of Sports, Culture and the Arts	Ministry of Interior and Coordination of National Government Ministry of Defence Ministry of Devolution and Planning Ministry of Finance & National Treasury
Environment and sustainability	Access to safe water	SDG 6: Ensure availability and sustainable management of water and sanitation for all SDG 7: Ensure access to affordable, reliable, sustainable and modern energy for all SDG 13: Take urgent action to combat climate change and its impacts SDG 14: Conserve and sustainably use the oceans, seas and marine resources for sustainable development SDG 15: Protect, restore and promote sustainable use of terrestrial ecosystems, sustainably manage forests, combat desertification, and halt and reverse land degradation and halt biodiversity loss	Ministry of Water & Irrigation Ministry of Environment, and Natural Resource Ministry of Mining	
Agriculture and food	Adequate nutrition	SDG 2: End hunger, achieve food security and improved nutrition and promote sustainable agriculture SDG 12: Ensure sustainable consumption and production patterns	Ministry of Agriculture, Livestock and Fisheries	
Housing and community services	Safe housing	SDG 11: Make cities and human settlements inclusive, safe, resilient and sustainable	Ministry of Land, Housing and Urban Development	
Economy and employment	Occupational hazards, unemployment	SDG 8: Promote sustained, inclusive and sustainable economic growth, full and productive employment and decent work for all	Ministry of Labour & East Africa Affairs Ministry of Industrialization and Enterprise Development Ministry of Foreign Affairs & International Trade Ministry of East Africa Affairs, Commerce, and Tourism	
Infrastructure and planning and transport	Road safety	SDG 9: Build resilient infrastructure, promote inclusive and sustainable industrialization and foster innovation	Ministry of Transport and Infrastructure Ministry of Energy and Petroleum	
Security and justice	Security	SDG 16: Promote peaceful and inclusive societies for sustainable development, provide access to justice for all and build effective, accountable and inclusive institutions at all levels	Ministry of Defence	
Land and culture	-	-	Ministry of Land, Housing and Urban Development Ministry of Sports, Culture and the Arts	
Multisectoral	SDG 1: End poverty in all its forms everywhere SDG 5: Achieve gender equality and empower women and girls SDG10: Reduce inequality within and among countries SDG 17: Revitalize the global partnership for sustainable development			

Abbreviation: SDG, Sustainable Development Goal. Notes: Sources include the Adelaide Statement on HiAP (column 1), Kenya Health Policy document 2014–2030 (column 2), SDGs document (column 3).

###  Assessing if HiAP Can Be Monitored and Reported Using SDGs in Kenya

####  OECD Building Block: Monitoring and Reporting HiAP Using SDGs Indicators 

 The OECD principle of monitoring and reporting proposes the need to identify and use targets and indicators to track progress.^35^Table 6 maps out the SDGs in relation to the specific sectors stated in the Adelaide Statement on HiAP and the social determinants of health highlighted in the Kenya Health Policy document. We used the Environment and Sustainability sector as an example to illustrate how the progress to attain access to safe water and adequate sanitation can be monitored using the SDG indicators.

 Safe water and adequate sanitation are essential to prevent outbreaks of water-borne diseases such as cholera which Kenya is prone to. SDGS 6, 7, 13, 14, and 17 are all relevant to this sector. Specifically, SDG 6 addresses availability and sustainable management of water and sanitation for all. Table 7 lists examples of relevant indicators under SDG 6.

**Table 7:** Examples of SDG 6 Indicators Relevant to Access to Safe Water and Nutrition

**SDGs**	6
**Social determinants of health**	Access to safe water
**SDG** **Indicators**	6.1.1 Use of safely managed drinking water services
	6.2.1 Use of safely managed sanitation services
	6.3.1 Safely treated wastewater
	6.3.2 Water bodies with good ambient water quality
	6.4.1 Efficiency in water usage
	6.4.2 Availability of fresh-water resources

Abbreviation: SDG, Sustainable Development Goal.

 The proportion of the Kenyan population using safely managed drinking water services in the urban area dropped from 51.7% in 2015 to 50.0% in 2017. The proportion of the population practicing open defecation as a component of safely managed sanitation services declined from 11.1% in 2015 to 10.3 % in 2017 in both rural and urban areas. The proportion of the population with basic hand washing facilities both in rural and urban areas remained constant between 2015 and 2017 at 24.6%. In 2017, 35.5% of the water bodies in Kenya had good ambient water quality. Water usage efficiency was recorded at $10.9 per cubic meter in 2015. Still in 2015, there was only 33.2% of fresh-water resources available.

 From the available data, Kenya is off-track ensuring access to safe water and adequate sanitation for all Kenyans by 2030.

####  OECD Building Block: Policy Effects

 Addressing a policy’s impact and effect is a key building block in facilitating PCSD^35^. Countries all over the world are realizing the importance of impact assessments to ensure sustainability of development policies.^35,64^ More specifically, health impact assessment (HIA) is recognized as one potentially powerful tool that can be used to support HiAP.^65^ Implementation of SDGs has been seen as an opportunity for countries to introduce HIA.^64^

 Interview statements were contradictory with some interviewees indicating that impact assessments are done occasionally, whereas others said they are always done. In terms of who conducts the assessments, we were met with an equal variety of responses including: private consultants, civil society organizations, National Environmental Management Agency (NEMA) etc. It is worth noting that NEMA is mandated to conduct Environmental Impact Assessments in Kenya, which some interviewees mentioned they use as a “*proxy*” for HIA. However, dissemination of NEMA recommendations is suboptimal as expressed by 3 interviewees.

*“NEMA will send us a report of Environmental Impact Assessments of a proposal with project of course it may be sent to health [Ministry of Health], [The reports] may be sent to Nairobi City County but again that synchronization is not there” *(K5 – Government Official).

 In summary, with availability of data, SDG goals and indicators could be used as a component of monitoring the progress of the sectors outlined in the Adelaide statement on HiAP. There is a need to recognize HIA as an essential tool to support HiAP.

## Discussion

 In the discussion, we reflect on the 3 aims we embarked on using the OECD’s building blocks for effective policy coherence in Kenya, as it related to HiAP and SDGs.

###  Understand How a HiAP Approach Can Leverage Development Agendas Promoting the SDGs

 Consistent with literature, we found that the HiAP approach faces challenges in moving from rhetoric to practice.^66,67^ Electoral cycles and political party preferences have often upended HiAP and SDG implementation thereby stressing the importance of instating more robust structures and processes for embedding HiAP into government policies.^35^ This can be done by explicitly including HiAP in the Vision 2030 similar to Namibia, Sudan, Zambia and Suriname who have used national development plans to successfully adopt HiAP.^68^ These countries did this as a way to give health a central focus on development as part of the whole-of-government approach.^68,69^ By explicitly stating that HiAP will be the core approach for Vision 2030, there will be a mutual benefit of visibly tracking the progress of in-country policy interventions to regional and global benchmarks.

###  Assess How Both the HiAP Approach and the SDGs’ Implementation Were:

####  a) Articulated in Kenyan Policy Documents 

 With respect to policy integration, the study found that Vision 2030 has aligned with Agenda 2030 and Agenda 2063, but not seamlessly. This was also the same finding by the SDG Kenya Forum’s 2016 analysis of these two agendas in relation to Kenya’s Vision 2030.^51^ In this report, the forum suggests that specific targets, as highlighted within the different agendas ought to be visibly outlined in implementation matrices, to allow for comparative monitoring of Kenya’s in-country progress of interventions and assess its ambitions towards meeting continental and global targets.^51^ In spite of the highlighted gaps, the forum experts deemed the existing processes as sufficient to support the country’s progress for achieving SDGs and Agenda 2063.^51^

####  b) How They Are Perceived by the Various Stakeholders

 Our study demonstrated that policy coordination and mapping of stakeholders’ engagement is at the core of propagating HiAP and SDGs, and assist with strengthening existing mechanisms for both horizontal and vertical co-ordination.^67,70^ However, “turf wars” do exist and without appropriate co-ordination mechanisms to allow ministries, public sector agencies and other key stakeholders to share information, define and distribute responsibilities and efficiently allocate resources for SDG implementation, HiAP as well as the SDGs will struggle to be realized. It will be imperative for Kenya to specifically state how inter-ministerial conflicts can be addressed.^71^ In this study we were able to give an example of how ministries could collaborate or take up leadership roles in facilitating ISC.

 Kenya also has both governmental and civil society bodies working on the SDGs, especially at national level. However, the implementation is at local level. Thus, the principle of subnational and local involvement is vital as there is an emphasis on these two levels working together in the 2030 agenda. Local governments have proven to be strategically positioned to harness partnerships between stakeholders and better gauge health priorities at community level.^72^ In Kenya, the HPACs could also align more clearly with the County level SDG structures and groups already present. Their role could be expanded to support oversight mechanisms. This will demonstrate how county-specific decisions and health resources can translate into positive health outcomes.^73^ HPACs could also collaborate more with both the national and local academic institutions and perhaps aim towards a knowledge translation platform role such as the Health Policy (not promotion) Advisory group in Nigeria.^74^ As SDGs have funds allocated both at the national and the county level, the HPACs in Kenya would benefit from aligning some specific plans alongside the SDG agenda. The county health directors and the finance departments that are being set up at the county level should also be lobbied to consider HiAP in their budgeting process.

###  Offer Insights into the Opportunity of Developing a Monitoring and Reporting Framework for Kenya’s HiAP Approach Using SDG Indicators

 On monitoring and reporting, we found out that SDG Indicators can be used to inform the progress for sectors outlined under HiAP. Many SDG indicators can be conceptualized as health determinants and HiAP is very essential in realizing policy coherence with the SDGs.^68^ We suggest that to demonstrate this coherence, a HiAP and SDG database could be developed under the given sectors that address the economic, social and political components of development. This could also be used as a basis to further streamline the HiAP priorities in a given county, including collaboration with specific stakeholders.

 The potential of the HIA to assess the policy effects of HiAP was explored at the 8^th^ International HIA Conference in Ireland in 2007.^75^ This has also been discussed by other multilateral and national agencies.^65,76^ St-Pierre considered HIA as one of the most structured approaches to adopting HiAP given its ability to better inform decision-makers outside of the health sector of the link between health and their given sectors.^76^ The exploration of HIA as a tool for HiAP and its implications or relation to the SDG indicators is an interesting area of research.

###  Pros and Cons of Using the OECD framework

 To the best of our knowledge, this is probably one of the first and very few studies that has rigorously applied the OECD framework for academic/scientific research. However, the OECD team did use empirical data in their report on this framework.^35^ The OECD policy framework principles were useful to assess empirical data on policy coherence between the SDGs and HiAP. Even though some principles were indeed represented in the data less saliently than others, including them in our findings report still enabled us to highlight interesting insights. In addition, they indicate areas where the OECD framework can be improved. It was also noted that there was no principle directly addressing funding which is an important influencer of policies in LMICs.

###  Study Limitations

 At the time of this study, both SDGs and HiAP were at very early adoption stages. As such, the data available was not adequate to derive extensive conclusions from. Due to time and financial constraints, interviews were only conducted with stakeholders at national level. Future qualitative studies on synergies between SDGs and HiAP should include local level key informants, documents and funding structures. We also recommended that future studies use the OECD framework as a *priori*, that is, to inform the development of the interview guide data collection (including the development of interview guides). Upon availability of SDG indicator data, the proposed HiAP and SDG indicator database can be further analyzed, improved and valuable comparisons among countries made.

## Conclusion

 Due to its devolved governance structure, Kenya has a unique opportunity to build momentum for HiAP alongside SDGs. This can be achieved by leveraging on the political commitment SDGs have at all government levels. The establishment of HPACs in collaboration with the SDG structures is a plausible step and running such structures through the devolved government could be important for the long-term sustainability of HiAP. HiAP adoption can also benefit from the long-term planning horizons of the SDGs which have been able to address the quick turn-over of political cycles. Funding for HiAP is crucial for its propagation, especially in LMICs and can be considered in the Budgetary allocations in the SDGs implementation. Institutions and stakeholders both from within and outside the government could collaborate to address policy coherence between HiAP and SDGs. Finally, to facilitate moving HiAP from rhetoric to action, HiAP can be monitored concurrently with the SDGs using the SDG indicators.

## Acknowledgements

 The first author acknowledges Paul and Maria Kremer Stiftung for funding her PhD work under which this study was undertaken. The authors would like to thank Dr. Catherine M. Jones for sharing her theoretical and conceptual reflections on policy coherence, which helped us identify the guiding framework for this paper.

## Ethical issues

 Ethical approval was obtained from the University of Nairobi and Kenya Medical Research Institute (KEMRI) Ethical Committee in Kenya (P472/06/2016) and the University of Heidelberg Ethical Commission (S-330/2016). A research permit clearance was also obtained from Kenya’s National Commission for Science, Technology and Innovation (Permit Number NACOSTI/P/17/83941/15349).

## Competing interests

 Authors declare that they have no competing interests.

## Authors’ contributions

 JM, LG, JT, and AJ developed the concept and design of the study. Data was collected by JM, LT, and JT and analyzed by JM, LT, and NS. The manuscript was drafted entirely by JM. All the authors participated in the critical revision of the manuscript for important intellectual content. AJ and JT were this paper’s and overall study’s supervisors.

## Authors’ affiliations

^1 ^Heidelberg Institute of Global Health, Heidelberg University, Heidelberg, Germany. ^2^Département de Gestion, Évaluation et Politique de Santé, École de Santé Publique, Université de Montréal, Montreal, QC, Canada. ^3^Department of Sociology, McGill University, Montreal, QC, Canada. ^4^Department of Family and Community Health, School of Public Health, University of Health and Allied Health Sciences, Ho, Ghana. ^5^Department of International Health, Johns Hopkins Bloomberg School of Public Health, Baltimore, MD, USA. ^6^Center for Evidence Based Health Care, Department of Global Health, Stellenbosch University, Stellenbosch, South Africa. ^7^Africa Centre for Evidence, University of Johannesburg, Johannesburg, South Africa. ^8^Institute of Political Science, Heidelberg University, Heidelberg, Germany.
